# Combined Laparoscopic and Posterior Approach Resection of Presacral Neuroblastoma with Rectobulbar Urethral Fistula

**DOI:** 10.70352/scrj.cr.25-0447

**Published:** 2025-11-07

**Authors:** Takashi Kobayashi, Yoshiaki Kinoshita, Junkichi Takemoto, Yuhki Arai, Yu Sugai, Koichi Saito, Shoichi Takano, Naoki Okuyama

**Affiliations:** 1 Department of Pediatric Surgery, Niigata University Graduate School of Medical and Dental Sciences, Niigata, Niigata, Japan; 2 Department of Pediatric Surgery, Niigata Prefectural Central Hospital, Joetsu, Niigata, Japan

**Keywords:** presacral tumor, retrorectal tumor, neuroblastoma, imperforate anus, rectobulbar urethral fistula, children, pediatric, laparoscopic surgery, laparoscope, outcome

## Abstract

**INTRODUCTION:**

We herein report a case of presacral neuroblastoma (NB) with a rectobulbar urethral fistula. We successfully resected the tumor with a combined laparoscopic and posterior approach and simultaneously performed posterior sagittal anorectoplasty (PSARP).

**CASE PRESENTATION:**

A 1-year-old boy underwent laparoscopic surgery. He had a surgical history of transverse colostomy for an imperforate anus (later diagnosed as a rectobulbar urethral fistula) on the 2nd day after birth. Before radical surgery for a rectobulbar urethral fistula at 1 year of age, an imaging study incidentally showed a 28 × 27-mm presacral tumor. After a detailed examination, the tumor was diagnosed as NB, International Neuroblastoma Risk Group (INRG) Stage L1. We decided to perform surgical resection using a combined laparoscopic and posterior approach. The main reason for using the laparoscopic approach was to reduce intraoperative bleeding by ligating the median sacral artery (tumor-feeding artery). We also planned to simultaneously perform PSARP. If PSARP is performed later, postoperative adhesions make it difficult to dissect the rectum and identify the levator ani muscles. Under general anesthesia, the median sacral artery was ligated laparoscopically. The patient was then placed in the jackknife position, and the tumor was completely resected using a posterior approach. PSARP was performed without complications. The pathological diagnosis was NB, a differentiating subtype with R0 resection. The final INRG risk classification was low-risk, and no additional treatments were required. Postoperative complications were not observed, with the exception of urinary incontinence. The patient was discharged on the 16th day after surgery. He had no recurrence for 3 years after surgery. His defecation was well controlled using glycerin enema without soiling. His self-catheterization for urinary incontinence once daily was continued for 1 year and stopped after confirming no residual urine.

**CONCLUSIONS:**

In this study, we performed laparoscopic surgery combined with a posterior approach for a presacral NB and successfully resected the tumor with a good laparoscopic view. Furthermore, we simultaneously performed PSARP for the rectobulbar urethral fistula following tumor resection. This approach may be one of the options for treating presacral NB associated with a rectobulbar urethral fistula.

## Abbreviations

COG
: Children’s Oncology Group
HVA
: homovanillic acid
IDRF
: image-defined risk factors
INPC
: International Neuroblastoma Pathology Classification
INRG
: International Neuroblastoma Risk Group
LDH
: lactate dehydrogenase
MIBG
: metaiodobenzylguanidine
NB
: neuroblastoma
NSE
: neuron-specific enolase
PSARP
: posterior sagittal anorectoplasty
VMA
: vanillylmandelic acid

## INTRODUCTION

NB is one of the most common extracranial solid tumors in children. Low-stage localized tumors with favorable biology can be cured by surgical resection alone.^1)^ There are 3 approaches to presacral tumors in the pelvic cavity: transabdominal, posterior, and a combination of both approaches. In recent years, laparoscopic surgery has been reported to be useful as a transabdominal approach for benign presacral tumors.^2)^

We herein report a case of presacral NB in a 1-year-old boy with a rectobulbar urethral fistula. We used a combined laparoscopic and posterior approach for tumor resection and simultaneously performed PSARP for the rectobulbar urethral fistula.

## CASE PRESENTATION

**Fig. 1:**
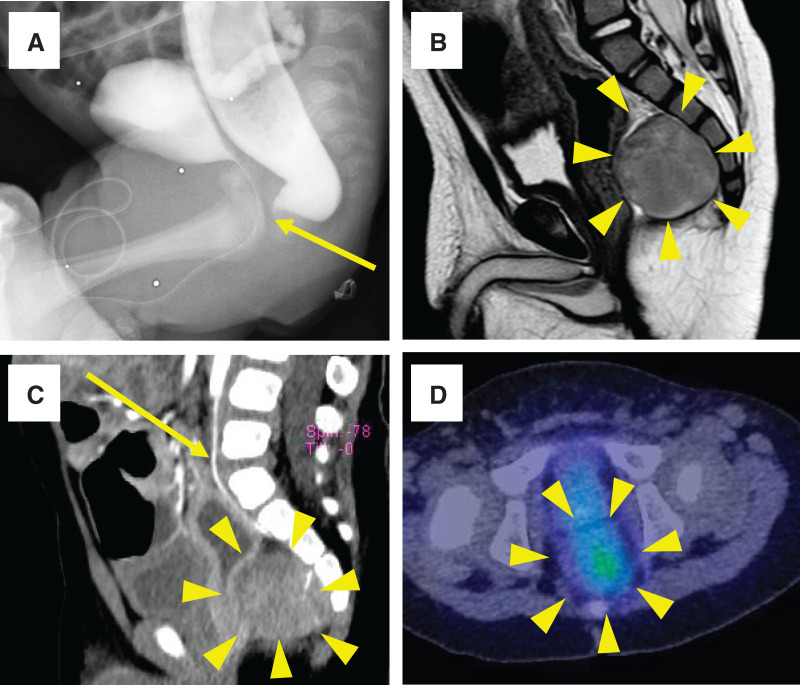
Imaging studies before surgery. (**A**) The imperforate anus was diagnosed as a rectobulbar urethral fistula (arrow) by voiding cystourethrography and colonorectography at 1 month of age. (**B**) Abdominopelvic MRI revealed a presacral tumor with a clear border and smooth surface. The tumor showed slightly high intensity on T2-weighted imaging (arrowheads). (**C**) Contrast-enhanced abdominopelvic CT showed that the tumor was a heterogeneously enhanced mass (arrowheads), and its feeding artery (arrow) was identified as the median sacral artery. (**D**) ^123^I-metaiodobenzylguanidine scintigraphy showed abnormal uptake in the intrapelvic presacral tumor (arrowheads).

A 1-year-old boy was admitted to Niigata University Hospital because of a presacral mass detected on pelvic MRI. He was born with an imperforate anus and underwent a transverse colostomy 2 days after birth at a local hospital. The imperforate anus was diagnosed as a rectobulbar urethral fistula using voiding cystourethrography and colonorectography at 1 month of age (**Fig. 1A**). No mass shadow or compression of the rectum was observed. A presacral tumor was not detected at 1 month of age. Pelvic MRI was performed to evaluate the levator ani muscles before PSARP for the rectobulbar urethral fistula. Incidentally, it revealed a 28 × 27-mm presacral tumor. The patient was then admitted to our hospital for further examination and treatment. On presentation, a physical examination revealed no palpable abdominal masses. Laboratory findings showed elevated LDH (366 U/L), NSE (35.0 ng/mL; reference range <16.3ng/mL), urinary VMA (37.5 mg/g·Cre; reference range <15.5 mg/g·Cre), urinary HVA (53.9 mg/g·Cre; reference range <27.3 mg/g·Cre). Colonorectography showed dorsal compression of the rectum and a mass shadow in the presacral space, which indicated the presence of a presacral tumor. Abdominopelvic MRI revealed a presacral tumor with a clear border and smooth surface. The tumor showed low intensity on T1-weighted imaging and slightly high intensity on T2-weighted imaging (**Fig. 1B**). Contrast-enhanced abdominopelvic CT showed a heterogeneously enhanced mass, and the median sacral artery was identified (**Fig. 1C**). ^123^I-MIBG scintigraphy showed abnormal uptake of the intrapelvic presacral tumor (**Fig. 1D**). Bone scintigraphy revealed no abnormalities in uptake. Whole-body CT showed no evidence of distant metastasis or IDRFs.^3,4)^ These results suggest that the preoperative diagnosis of the presacral tumor was the INRG Stage L1.^4)^ Currarino syndrome^5)^ was excluded because there were no sacral anomalies. We planned to perform surgical resection with a combined laparoscopic transabdominal and posterior approach because laparoscopic ligation of the median sacral artery was advantageous for reducing blood loss. We also planned to perform a presacral tumor resection and PSARP simultaneously. If PSARP is performed later, postoperative adhesions make it difficult to dissect the rectum and identify the levator ani muscles. To avoid this, we decided to perform simultaneous presacral tumor resection and PSARP.

### Surgical procedures

**Fig. 2:**
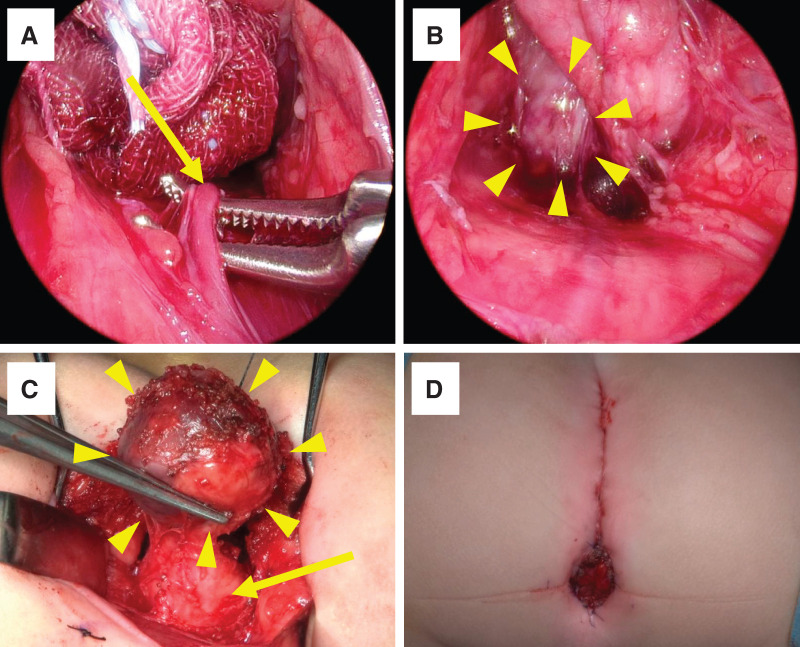
Surgical procedure and findings. (**A**) The median sacral artery was identified as the tumor-feeding artery. It was carefully isolated and laparoscopically ligated (arrow). (**B**) The presacral tumor (arrowheads) was dissected cranially and dorsally from the surrounding tissues. (**C**) The tumor (arrowheads) was almost fully exposed, except for the connective tissue between the tumor and the rectum (arrow). (**D**) Final anal appearance after surgery.

**Fig. 3:**
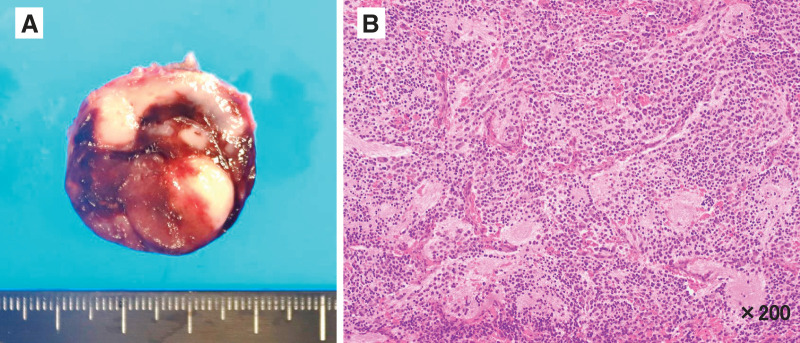
Macroscopic and microscopic findings of the excised specimen. (**A**) The tumor measured 29 × 25 × 22 mm in size, and no capsular injury was observed macroscopically. (**B**) Hematoxylin and eosin-stained sections at ×200 magnification show neuroblastoma, differentiating subtype.

First, laparoscopic surgery was performed. Under general anesthesia, the patient was placed in the supine position. The median sacral artery was identified as the tumor-feeding artery. It was carefully isolated and laparoscopically ligated (**Fig. 2A**). The presacral tumor was then dissected from the cranial and dorsal parts of the surrounding tissues (**Fig. 2B**). After temporary closure of the wound at the abdominal port site, the patient was placed in the jackknife position. A posterior sagittal incision was made, and the coccygeal bone was removed. The tumor was exposed and isolated along the sacral bone, and the dissection plane was connected to the abdominal laparoscopic approach layer. The tumor was almost completely exposed, except for the connective tissue between the tumor and the rectum (**Fig. 2C**). Finally, the presacral tumor was completely resected without any rectal injury. PSARP was then performed for the rectobulbar urethral fistula using the same posterior approach incision and was completed without intraoperative complications (**Fig. 2D**). After closure of the posterior approach wound, the patient was returned to the supine position, and laparoscopic surgery was restarted. We confirmed the absence of intestinal twisting or intra-abdominal bleeding laparoscopically. It took 154 min for skin incision and laparoscopic surgery, 58 min for the supine-to-jackknife position change, 305 min for tumor resection and PSARP, 21 min for the jackknife-to-supine position change, and 53 min for laparoscopic surgery again and abdominal closure. The operative time was 591 min, with 20 mL of blood loss and no blood transfusion. The tumor measured 29 × 25 × 22 mm in size, and no capsular injury was observed macroscopically (**Fig. 3A**). The histopathological diagnosis was NB, differentiating subtype, and a low Mitosis-Karyorrhexis Index with R0 resection (**Fig. 3B**). The INPC showed a favorable histology. *MYCN* amplification was not observed in this study. The INRG risk classification was low-risk, and no additional treatments were required.^6)^ Postoperative complications were not observed, with the exception of urinary incontinence. The patient was discharged on the 16th day after surgery. During follow-up in the outpatient clinic, the patient’s defecation was well controlled with a daily glycerin enema. No soiling or other complications were noted. His self-catheterization for urinary incontinence once daily was continued for 1 year and stopped after confirming no residual urine. The patient had no evidence of NB recurrence at 3 years after surgery.

## DISCUSSION

We herein report a rare case of presacral NB with a rectobulbar urethral fistula. We successfully resected the presacral tumor using a laparoscopic transabdominal approach combined with a posterior approach. The laparoscopic approach was useful for minimizing bleeding by the 1st ligation of the tumor-feeding artery. The combination of laparoscopic and posterior approaches was also useful for maintaining a clear view during tumor resection. We safely performed tumor resection and PSARP. This is the 1st case report of presacral NB combined with a rectobulbar urethral fistula.

NB is characterized by its biological diversity, and it is important to select treatment based on risk classification according to age, stage, *MYCN* gene amplification, and other features.^6)^ Surgical treatment was selected based on the risk group. IDRFs are important in determining whether a tumor is resectable or not. IDRFs are used to evaluate localized NB cases, which estimate the risk of surgery from imaging findings and determine whether to attempt complete resection or biopsy as an initial surgery.^4)^ In this case, the imaging was negative for IDRFs, and the tumor was localized in the presacral space. The clinical stage was L1, and surgical resection was performed. Because the COG risk was classified as low-risk after surgery, no additional treatment was necessary, and the patient was followed up.^7)^

Laparoscopic surgery for NB has only been performed in a limited number of cases, and there is insufficient evidence regarding its usefulness.^8,9)^ In Japan, the 1st case of laparoscopic adrenectomy for NB was reported in 1996.^10)^ Since then, although reports of laparoscopic surgery have been increasing, they still account for only about 12% of cases in a nationwide survey in Japan.^11)^ However, evidence of laparoscopic surgery for NB is gradually increasing. A systematic review by the American Pediatric Surgical Association reported that minimally invasive surgical resection of NB is safe for carefully selected cases (those <4–6 cm in size and abdominal tumors that are negative for IDRFs).^12)^ Based on a multicenter study, the International Society of Paediatric Oncology Europe—Neuroblastoma (SIOPEN) has proposed guidelines for minimally invasive surgical resection for NB, which state that small, localized NBs without IDRFs and with a tumor volume of <75 mL are suitable for laparoscopic resection, and those with a tumor volume of <60 mL and only 1 IDRF may also be considered for laparoscopic resection.^13)^ Sugita et al. reported that if cases are selected appropriately, avoiding difficult cases such as those with IDRF positivity, the time to start oral nutrition is sooner, and the operation time is shorter in laparoscopic surgery cases than in open surgery cases.^14)^ In our case, laparoscopic surgery was effective in identifying and safely ligating the median sacral artery with a clear magnified view. We believe that laparoscopic surgery contributes to the minimization of blood loss.

Traditionally, there are 3 approaches to presacral tumors: the transabdominal, posterior, and a combination of both approaches. The approach was selected based on the location and size of the tumor. Generally, if the tumor is above the 3rd sacral vertebra, an abdominal approach is used; if the tumor is below the 3rd sacral vertebra, a posterior approach is used. A combined approach is used for other tumors above and below the 3rd sacral vertebra.^15)^ The disadvantages of the posterior approach are a higher risk of intraoperative bleeding and injury to the lateral pelvic nerves because of poor visualization of the cranial side of the tumor.^16)^

In 1995, the 1st laparoscopic transabdominal approach for presacral tumors was reported.^17)^ Laparoscopic surgery is useful for accurately understanding anatomical structures because of its clear magnified view. It helps to avoid damage to the pelvic muscles and pelvic nerves and is useful for controlling vessel bleeding, especially from the median sacral artery.^18,19)^ Minimizing incisions may also reduce postoperative wound pain and infection. A review of 83 cases of laparoscopic surgery reported that hospital stays were shorter than those for open surgery (4 ± 2 vs. 9 ± 7 days, p < 0.05), and the complication rates were 19.8% for laparoscopic surgery and 12.2% for open surgery, with no significant difference.^16)^ According to a multicenter study of 270 cases by Aubert et al., there was no difference in mortality, morbidity, reoperation, or readmission rates between laparoscopic surgery and the posterior approach.^20)^

There are several reports about combined vs. single (laparoscopic or posterior only) approaches in the treatment outcomes of presacral tumors. Li et al. reported that the combined approach was performed for larger tumors (median 12.85 cm) and showed a longer operative time (median 429.5 min) than the single approach (abdominal or posterior only). There was no difference in length of hospital stay, recurrence rate, and survival rate between the combined and single approaches.^21)^ Tsarkov et al. reported that the combined approach had a higher presacral cyst capsule rupture rate (45%) than the posterior approach (24%, p = 0.011), although it was not different from the laparoscopic approach (61%). There was no difference in surgical complications between combined and single (laparoscopic or posterior) approaches.^22)^

Unfortunately, there have been no reports of simultaneous presacral NB resection and PSARP for imperforate anus. However, there have been reports of presacral tumor resection and PSARP for the treatment of Currarino syndrome.^23)^ AbouZeid et al. reported a 3-month-old boy with a presacral tumor and imperforate anus associated with Currarino syndrome. In this case, excision of the presacral cyst was performed using a combined approach in 2 steps: the 1st excision during the PSARP procedure in the prone position, and the 2nd operation in the supine lithotomy position to remove a residual component of the lesion that was missed during the primary operation. They concluded that the combined approach was useful for deep presacral tumor resection.^24)^ We also added a literature review of case reports on PSARP for imperforate anus and presacral tumor resection in **Table 1**.

**Table 1:** Literature review of case reports of PSARP for imperforate anus and presacral tumor resection

	Author	Publication year	Age at surgery	Sex	Symptoms	Currarino syndrome	Management	Excised tumor size (mm)	Histological type of tumor	Postoperative hospital stay (days)	Follow-up (months)	Outcomes
1	Lee et al.^23)^	1997	29 months	M	Constipation	Yes	PSARP, tumor excision (posterior approach)	N/A	Teratoma	N/A	36	N/A
2	Lee et al. ^23)^	1997	1 day	F	Absence of anus	Yes	PSARP, tumor excision (posterior approach)	N/A	Teratoma	N/A	21	N/A
3	AbouZeid et al.^24)^	2019	3 months	M	Absence of anus	Yes	PSARP, tumor resection (metachronous combined open abdominal and posterior approach)	20	Developmental cyst	N/A	3	Spontaneous defecation once every 1–2 days
4	Present study	2025	1 year	M	Absence of anus	No	PSARP, tumor resection (simultaneous combined laparoscopic and posterior approach)	29 × 25 × 22	Neuroblastoma	16	36	No tumor recurrence, controlled defecation with daily glycerin enema, temporarily (1 year) urinary incontinence

F, female; M, male; N/A, not available; PSARP, posterior sagittal anorectoplasty

In surgery for pelvic NB, there is a certain rate of urinary and bowel dysfunction due to the tumor involving the pelvic visceral nerves. Previous studies have reported complication rates of 15%–35% after resection of pelvic NB, primarily related to urinary and fecal incontinence after injury to the sacral nerve roots.^25,26)^ In this case, the patient showed temporary postoperative urinary incontinence that disappeared 1 year after surgery. Although the bowel function depended on glycerin enema, defecation was well controlled without soiling.

## CONCLUSIONS

In this study, we performed laparoscopic surgery combined with a posterior approach for a presacral NB that was incidentally discovered during MRI prior to radical surgery for a rectobulbar urethral fistula and successfully resected the tumor with a good laparoscopic view. Furthermore, we simultaneously performed PSARP for the rectobulbar urethral fistula following tumor resection. This is the 1st report of a case in which presacral NB was associated with a rectobulbar urethral fistula. This may be one of the options for treating presacral NB associated with a rectobulbar urethral fistula.

## DECLARATIONS

### Availability of data and materials

The data that support the findings of this study are available from the corresponding author upon reasonable request.
